# A customizable secure DIY web application for accessing, sharing, and browsing aggregate experimental results and metadata

**DOI:** 10.1093/bioadv/vbae087

**Published:** 2024-06-28

**Authors:** Jaewoo Lee, Mehita Achuthan, Lucas Chen, Paulina Carmona-Mora

**Affiliations:** College of Engineering, University of California at Davis, Davis, CA 95616, United States; College of Engineering, University of California at Davis, Davis, CA 95616, United States; College of Engineering, University of California at Davis, Davis, CA 95616, United States; Department of Neurology, School of Medicine, University of California at Davis, Sacramento, CA 95817, United States

## Abstract

**Summary:**

A problem spanning across many research fields is that processed data and research results are often scattered, which makes data access, analysis, extraction, and team sharing more challenging. We have developed a platform for researchers to easily manage tabular data with features like browsing, bookmarking, and linking to external open knowledge bases. The source code, originally designed for genomics research, is customizable for use by other fields or data, providing a no- to low-cost DIY system for research teams.

**Availability and implementation:**

The source code of our DIY app is available on https://github.com/Carmona-MoraUCD/Human-Genomics-Browser. It can be downloaded and run by anyone with a web browser, Python3, and Node.js on their machine. The web application is licensed under the MIT license.

## 1 Introduction

Preparing data for analysis involves retrieving processed data, research results, and metadata. This is particularly applicable in writing processes (such as manuscripts, grant applications, or other documents). Typically, working data is in the form of individual tables (e.g. CSV files) summarizing results and metadata. Therefore, a common way to access it is through system browsing and retrieving different working sheets across several files. Protecting the access to the storage and sharing of this data is essential, as processed data and working results should maintain laboratory-specific identifiers to facilitate analyses. Scattered data poses a hindrance to efficient research. Our DIY web application allows users to upload datasets or processed results (CSV files), view and edit dataset information, link information to open databases, manage team users and permissions, and bookmark specific information and datasets. It ensures data security with a permission system to prevent unauthorized access or changes. The UI (User Interface) is intuitive and allows easy access to extract information for other research processes (such as grant or publication writing).

REST API (Representational State Transfer Application Programming Interface) calls to the backend to update the UI based on user actions, including searching, editing the data, undoing edits, and deleting. The data architecture includes various objects, like users, datasets, data features, samples, etc. The information in the file is converted into objects and objects are linked using names. Displaying a table in the frontend based on the associated objects is handled by the web application. External APIs, like NCBI (Sayers *et al.* 2022), bioDBnet (Mudunuri *et al.* 2009), and Ensembl REST API (Yates *et al.* 2015, Martin *et al.* 2023), were used to obtain different feature information, in this case gene name, mRNA code sequence segments, assembly information, type of gene, etc. The frontend, backend, and external APIs together allow users to view, delete, edit, and upload processed data, results, and associated online information on a common platform. Besides providing easy access to research results, this web application can also enhance collaboration within a laboratory, while the code provides customizable options to tailor features to specific uses.

## 2 Architecture of systems

**Figure 1.**
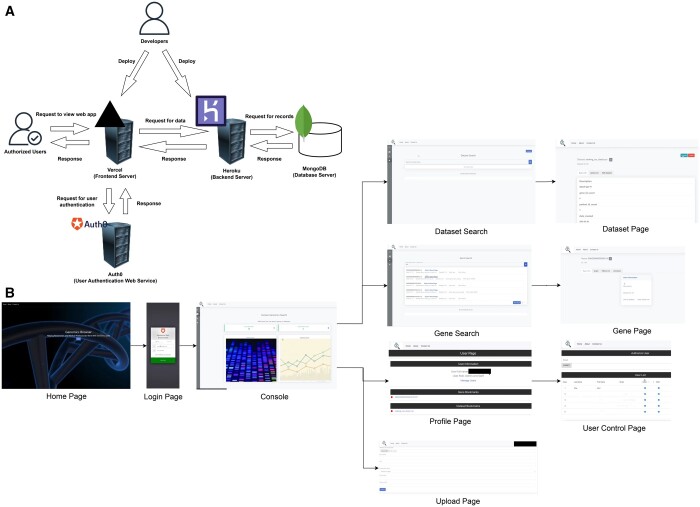
(A) Architecture overview. The main technology stacks of this web application are: React, Django, Auth0, MongoDB. React was used to implement the frontend and JavaScript to write the code. Django was used for the backend to create a connection between the frontend and the database. Any communication of data or processing of data is done using Django. MongoDB was used for the database, and NoSQL database was chosen for storage flexibility. Auth0 was used as an authentication system. (B) Application path. View of all the pages that exist in the sample web application and how users can move between those pages.

The architecture tiers used to develop this application are discussed here and presented in Fig. 1A.

### 2.1 Authentication

In order to use the web application, users must have an account to log into the system. Any user can create an account, but access to all pages is denied if the user's email is not registered in the system, as enabled by Auth0 (user management system) and MongoDB (email verification of registered user).

### 2.2 Uploading datasets

The upload process starts upon the user submission of the file on the upload page. From the frontend, the application attempts to send the CSV file to the backend server. If it fails for any reason, it will notify the user through the browser. When the backend server receives the file, the parsing process begins. During parsing, the dataset undergoes cleanup such as omitting rows or columns with “NA” (not available) values. Then, the identifier words given by the user when uploading the data are used to identify the type of the dataset (i.e. feature—in this case, gene ID dataset or sample dataset). Finally, using pandas (a Python data manipulation library) (McKinney 2010, The Pandas Development Team 2023), the server creates a list of data objects to be submitted to the MongoDB collection. The information of the dataset itself is submitted to the database. The ID is used to link the list and the dataset, allowing the removal of any feature or subject data when the dataset is deleted from the database.

### 2.3 Searching data

The search function (for ID or dataset) sends the keyword to the backend server, which will perform a fuzzy search algorithm to display engine-like functionality, returning results with a similar name to the keyword entered.

### 2.4 Tables in dataset/gene pages

In each dataset or gene (feature) page, data can be previewed, which is fetched when the user accesses that page. The table edit functionality does not upload the edits every time the user makes one. Instead, all changes are sent to the backend collectively. Once updated, the information is fetched and the table preview will be edited on top of the original data, being stored for each dataset in a user-independent manner.

### 2.5 Bookmark

Bookmarking is possible at feature and dataset levels, and this information is stored in MongoDB for each user. After selecting the bookmark button, the page’s ID is sent to the backend, triggering a request to MongoDB to store that ID information to the list in that specific user's information in MongoDB.

## 3 Additional APIs used

Although not primarily used, some external APIs were used to enrich the quality of the web application. NCBI DB (Sayers *et al.* 2022) was used to translate gene ID (feature ID) to its name. The API provided by GeneCards (Stelzer *et al.* 2016, Safran *et al.* 2021) creates an URL leading to a GeneCards page with search results for a specific gene. For visual enhancement, the web application displays colored segments of mRNA code. This was achieved using the bioDBnet API (Mudunuri *et al.* 2009) to convert the ENSEMBL gene ID (Yates *et al.* 2015, Martin *et al.* 2023) to the RefSeq mRNA Accession identifier, which was used to make an NCBI API call to fetch the mRNA code segment for a gene. Extra gene data (assembly information, type, and other identifiers/names) were fetched with the ENSEMBL API.

## 4 Functionalities

This web application enables the users to browse datasets, results, browse features (in this case gene data and gene information), search, edit datasets, and compare gene results (values) within a dataset (Fig. 1B). Initially designed to browse and share genomics results and metadata, the web application can be customized for other projects (a detailed description of each existent functionality is in Supplementary Information).

## 5 Limitations

Although the current version has stable functionalities, the scalability of the web application could be refined to allow more requests, as currently the frontend server and the backend server are directly connected. Multiple backend servers and load balancing could be implemented to distribute the requests for improving the durability of the system.

## 6 Applications

Our work provides a new centralized processed/working data and metadata storage and sharing platform for research laboratories or teams, offering an alternative to the common practice of using third-party storage services like OneDrive or Google Drive. This DIY customizable and secure (restricted access) web application facilitates the comparison of diverse data, results, and sample lists, by generating easily searchable and navigable pages, thereby eliminating the need to access multiple sources regularly. Additionally, users can add metadata and comments, while uploading data and results, which can assist for organization and analysis purposes. The base code provided in the repository can be reproduced to deploy the app in the current form with the features shown in the video demonstration of the repository, or customized for other purposes, by linking different APIs.

## Supplementary Material

vbae087_Supplementary_Data
