# Supplementary material for: A customizable secure DIY web application for accessing, sharing, and browsing aggregate experimental results and metadata
Source: Bioinform Adv. 2024 Jun 28;4(1):vbae087. doi: 10.1093/bioadv/vbae087 (PMC11257709; doi:10.1093/bioadv/vbae087)
Supplement: vbae087_Supplementary_Data [file vbae087_supplementary_data.docx]

**Supplementary Information**

# **Detailed functionalities**

# The main functionalities are described using our gene browser web application as an example. The functionalities are divided into pages of the web application.

**Home page:** This is the initial view for users, visible before logging in. From this page, there are buttons through which users can reach the About Page (purpose of the web application) and Contact Page (relevant contact information).

**Login/Signup (user):** Users can create a new account or login using their account credentials. Anyone can create an account, but not every user can access all the resources. The login system was implemented since the purpose of the system is to share, store and analyze working data and results, which have project and internal identifiers in a working group. Additionally, access to viewing and manipulating data is restricted to authorized users.

**Main Console Page:** First landing page for the logged in user (previously registered by the admin, if not verified, the user is directed to a page that informs the need of admin verification). Users access all the functionality of the application with intuitive navigation, with buttons to search pages for genes or datasets. The page also displays the count of stored genes and datasets.

**Upload:** Admin-verified users can upload data (CSV format). Clicking the upload button transfers all data to the backend server, where necessary work, such as parsing the data, is done. Metadata can be entered at this point, such as description of dataset, link to a shared drive, etc. Some of the metadata is used for parsing, for example, in the sample gene database, sample or gene ID were used for the identification of the sample or gene columns during parsing; these values must be valid. There is error checking for this in the backend during parsing. Additionally, the dataset name is used for checking duplicated uploads by other users. Metadata can be edited if necessary.

**Gene search:** This page displays a search bar for typing keywords and can be accessed from the Main Console page. There is a drop-down selection that allows selecting the search fields, such as “Gene Name” or “Other Name” (alternative name for the Gene other than the Ensembl ID). The search results are fetched or refreshed after pressing the search button. This search is fuzzy, therefore there will be some ranking based on similarity. The results will have some basic information and a clickable link to the respective Gene Pages for the genes. There may be multiple entries for genes if multiple datasets have the same gene. At the bottom of the Gene Search Page, there is a scrollable box with bookmarked genes using a card layout, with a clickable link and brief gene information.

**Gene page:** Each gene (or row in the CSV file) has its own page. A Gene Page displays information related to a specific gene with a tab layout. There are multiple tabs, and each contains a different group of information. Tabs include “Basic Info”, “Graph”, “Patients (or sample) Info”, and “Code Sequence”. The top of the page shows the name of the gene and a clickable bookmark icon, allowing users to bookmark the gene. Bookmarked genes show up in the Gene Search page’s scrollable box and in the user profile page. The “Basic Info” tab may pull information from an external source, if possible, but it will list information about the gene from the dataset, except gene IDs and gene values. In our case, we included a link to GeneCards (Stelzer *et al.* 2016; Safran *et al.* 2021) with search results related to this gene. The “Graph” tab shows plots of the gene values, offering two graph types. The “Patients (sample) Info” tab shows a table of samples and the gene values associated with the gene searched per sample. It is possible to filter the table based on different column values. The table or graphs are not editable, and they can be empty if the dataset has no samples.

**Dataset search:** This page displays a search bar for typing keywords, with search results fetched or refreshed after pressing the search button. The search is fuzzy, so there will be some ranking based on similarity. An external package with functions related to fuzzy search was used in the backend. The results will have basic information and a clickable link to the respective Dataset pages for the datasets. At the bottom of the Dataset Search Page, there is a scrollable box with bookmarked datasets. The bookmarked datasets use a card layout, with a clickable link and brief dataset information.

**Dataset page:** At the top, the Dataset Page has the title of the Dataset, date uploaded, and the bookmark icon. Tabs include “Basic Info”, “Genes Info”, and “Dataset Table”. The “Basic Info” tab has information like the number of genes, samples, etc. The “Genes Info” tab has a list of links corresponding to genes in this dataset. The list is in a single-column table, and it is possible to filter values. The “Dataset Table” tab has an editable table with all the samples and genes information. Users can view the last five edits for the dataset and undo any of them. It is possible to delete a dataset with a button click.

**User page:** This page allows users to check account/personal information, including their name, role, and bookmarked pages (mentioned in the next subsection). A logged in user can check their own page only.

**Bookmark:** Users can click the bookmark part on either the gene page or the dataset page and save the page for later use. Bookmarked pages are listed on the User Page (for the respective user) and can be accessed and unbookmarked from there.

**User Management Page:** This page can be accessed by users with the Admin role. It allows the Admin to add users by registering emails, delete users, and change their permissions. It also displays a history log of user permission changes to allow the Admin to check for any unexpected or odd modifications.
